# Aged garlic extract preserves beta-cell functioning via modulation of nuclear factor kappa-B (NF-κB)/Toll-like receptor (TLR)-4 and sarco endoplasmic reticulum calcium ATPase (SERCA)/Ca^2+^ in diabetes mellitus

**DOI:** 10.1186/s13098-024-01350-8

**Published:** 2024-05-22

**Authors:** Sofi Imtiyaz Ali, Ahmed M. E. Elkhalifa, Showkat Ul Nabi, Faisal Sualeh Hayyat, Mehak Nazar, Syed Taifa, Rabia Rakhshan, Iqra Hussain Shah, Muzaffer Shaheen, Imtiyaz Ahmad Wani, Umar Muzaffer, Ovais Shabir Shah, Dil Mohammad Makhdoomi, Elsadig Mohamed Ahmed, Khalil A. A. Khalil, Elsharif.A. Bazie, Khalid Ibrahim Zawbaee, Moataz Mohamed Al Hasan Ali, Rakan J. Alanazi, Ibrahim Ali Al Bataj, Saeed Musfar Al Gahtani, Ali Jubran Salwi, Lina Saeed Alrodan

**Affiliations:** 1 Preclinical Research Laboratory, Department of Clinical Veterinary Medicine, Ethics and Jurisprudence, Faculty of Veterinary Sciences and Animal Husbandry, Sher-E-Kashmir University of Agricultural Sciences and Technology (SKUAST-Kashmir), Srinagar, Jammu and Kashmir 190006 India; 2 Department of Public Health, College of Health Sciences, Saudi Electronic University, 11673 Riyadh, Saudi Arabia; 3 Department of Haematology, Faculty of Medical Laboratory Sciences, University of El Imam El Mahdi, Kosti, 1158 Sudan; 4 Srinagar Women’s College, Zakura, Srinagar, Jammu and Kashmir 190024 India; 5 Department of Clinical Biochemistry, University of Kashmir, Srinagar, Jammu and Kashmir 190006 India; 6 Department of Endocrinology and Clinical Research, Sher-I-Kashmir Institute of Medical Sciences, Srinagar, Jammu and Kashmir 190002 India; 7 Department of Medicine, Govt. Medical College, Srinagar, Jammu and Kashmir India; 8 Department of Sheep Husbandry, Srinagar, Jammu and Kashmir 190006 India; 9 Directorate of Extension, Sher-E-Kashmir University of Agricultural Sciences and Technology (SKUAST-Kashmir), Srinagar, Jammu and Kashmir 190006 India; 10 Department of Medical Laboratory Sciences, College of Applied Medical Sciences, University of Bisha, 61922 Bisha, Saudi Arabia; 11 Pediatric Department, Faculty of Medicine, University of El Imam El Mahdi, Kosti, 1158 Sudan; 12 Department of Blood Bank, Autonomous University of Barcelona, Al-Ghad International College for Applied Sciences, 155166 Riyadh, Saudi Arabia; 13 Department of Pathology, Faculty of Medicine, Al-Baha University, Al-Baha, Saudi Arabia; 14 Department of Pathology, Faculty of Medicine, University of El Imam El Mahdi, Kosti, 1158 Sudan; 15 Department of Pharmacy Practice, College of Pharmacy, Alfaisal University, 50927 Riyadh, Saudi Arabia; 16 Ministry of Health, Najran General Hospital, 66262 Najran, Saudi Arabia; 17 Department of Blood Bank, College of Applied Medical Sciences, University of King Saud, 11433 Riyadh, Saudi Arabia

**Keywords:** Diabetes mellitus, Aged garlic extract, Streptozotocin, NF-κB/TLR-4, SERCA/Ca^2+^, Pancreatic β-cells

## Abstract

**Background:**

Peripheral insulin resistance and compromised insulin secretion from pancreatic β-cells are significant factors and pathogenic hallmarks of diabetes mellitus (DM). NF-κβ/TLR-4 and SERCA/Ca^2+^ pathways have been identified as potential pathways regulating insulin synthesis by preserving pancreatic β-cell functioning. The current study aimed to evaluate the therapeutic effect of aged garlic extract (AGE) against DM in a streptozotocin (STZ)-induced rat model with particular emphasis on pancreatic β-cell functioning.

**Methods:**

AGE was characterized by gas chromatography-mass spectrometry (GC-MS), Fourier-transform infrared spectroscopy (FTIR), and scanning electron microscopy (SEM) to evaluate its physio-chemical characteristics followed by in-vitro anti-diabetic and antioxidant potential. This was followed by the induction of DM in laboratory animals for investigating the therapeutic action of AGE by evaluating the role of NF-κβ/TLR-4 and the SERCA/Ca^2+^ pathway. The parameters assessed in the present experimental setup encompassed antioxidant parameters, metabolic indicators, insulin concentration, intracellular calcium levels, apoptotic markers (CCK-8 and Caspase Glo-8), and protein expression (P-62 and APACHE-II).

**Results:**

AGE characterization by SEM, GC-MS, and X-ray diffraction (XRD) revealed the presence of phenylalanine, alliin, S-allylmercaptocysteine (SAMC), tryptophan, 1-methyl-1,2,3,4-tetrahydro-β-carboline-3-carboxylic acid as major bioactive constituents of AGE. Metabolic studies, including intraperitoneal glucose tolerance test (IPGTT), revealed significantly lower blood glucose levels in the AGE group compared to the disease control group. In contrast, the intraperitoneal insulin tolerance test (ITT) exhibited no significant difference in insulin sensitivity between the AGE supplementation group and the DM control group. Interestingly, AGE was found to have no significant effect on fasting glucose and serum insulin levels. In contrast, AGE supplementation was found to cause significant hypoglycaemia in postprandial blood glucose and insulin levels. Importantly, AGE causes restoration of intracellular Ca^2+^ levels by modulation of SERCA/Ca^2^ functioning and inhibition NF-κB/TLR-4 pathway. AGE was found to interact with and inhibit the DR-5/ caspase-8/3 apoptotic complex. Furthermore, microscopic studies revealed degeneration and apoptotic changes in pancreatic β-cells of the DM control group, while supplementation of AGE resulted in inhibition of apoptotic pathway and regeneration of pancreatic β-cells.

**Conclusion:**

The current study suggests that AGE enhance glucose homeostasis by exerting their effects on pancreatic β-cells, without ameliorating peripheral sensitivity. Moreover, AGEs promote an increase in β-cell mass by mitigating the apoptosis of pancreatic β-cells. These findings suggest that AGE could aid in developing a viable alternative therapy for diabetes mellitus (DM).

## Introduction

Diabetes mellitus (DM) poses a significant health challenge impacting more than one billion people worldwide, and the metabolic syndrome is characterized by absolute hyperglycemia in response to insulin deficiency [1]. The global prevalence of DM has steadily increased over the past few decades. As per the International Diabetes Federation (IDF), approximately 537 million adults aged 20–79 years were living with diabetes worldwide in 2021. This number is expected to rise to 784 million by 2045[2, 3]. DM has multiple etiological factors, with environmental and genetic factors prominent in its pathogenesis [4]. The major etiological factors identified for DM include genetic predisposition [5], sedentary lifestyle [6], insulin resistance[7, 8], autoimmune destruction of β cells[8, 9], and pregnancy-related factors[9, 10]. A Plethora of studies has identified the key role of pancreatic β-cells in the pathogenesis of DM and the interplay between environmental and genetic determinants, resulting in the classical clinical manifestation of DM [11]. These studies have postulated that under-secretion of insulin, impaired functioning, and apoptosis of pancreatic β-cells play prominent roles in manifesting DM [12, 13]. Although various studies have identified the role of genomic determinants like the glucose transporters (GLUTs) family of receptors involved in the pathogenesis of DM, appropriately tailored therapies, have been designed to target these genomic determinants [14]. Instead, other unexplored determinants remain yet to be established, and their pathogenic role is poorly understood. Henceforth, an understanding of the molecular pathways that drive pathological β-cell defect involved in the pathogenesis of T2DM remains yet to be established [15].

In this direction recently, researchers have identified nuclear factor kappa-B (NF-κB)/Toll-like receptor (TLR)-4 and sarco endoplasmic reticulum calcium ATPase (SERCA)/Ca^2+^ pathways involved in the pathogenesis of DM [16]. Earlier studies have reported that various pathological pathways mediate peripheral insulin resistance without affecting insulin sensitivity. Nevertheless, few studies have reported that NF-κβ/TLR-4 and SERCA/Ca^2+^ pathways lower pancreatic β cell proliferation/insulin secretion in response to hyperglycemia [17]. Recently, dysregulation of NF-κβ/TLR-4 and SERCA/Ca^2+^ pathways in the Chinese population has been found to have an association with poor glycaemic control, increased prevalence of Polycystic ovarian syndrome (PCOS) and has been postulated to play a pivotal role in cold adoption during the evolutionary process [18]. These findings indicate the role of NF-κβ/TLR-4 and SERCA/Ca^2+^ pathways in energy homeostasis and demand further research to identify pathogenic hotspots activated by NF-κβ/TLR-4 and SERCA/Ca^2+^ pathways. Studies on the physiological and pathological role of NF-κβ/TLR-4 and SERCA/Ca^2+^ pathways are limited; some preliminary results have indicated that over-expression of NF-κβ/TLR-4 and SERCA/Ca^2+^ pathways causes hyperphagia in fruit flies which subsequently causes reduced thermogenesis and increases lipogenesis which culminates into obesity and reduced insulin resistance [19]. The pathogenic pathway postulated by these studies involves the inhibition of ATP biosynthesis and dissipation of energy in thermogenesis [20].

Similarly, preclinical studies have reported that the over-activation of NF-κβ/ TLR-4 and SERCA/Ca^2+^ pathways results in cold sensitivity and a role in the interaction of apoptosis proteins [21, 22]. Nevertheless, the physiological role of NF-κβ/ TLR-4 and SERCA/Ca^2+^ pathways in mammals remains yet to be established, and a minimal number of studies have reported its role in metabolic derangements. An in-depth understanding of NF-κβ/TLR-4 and SERCA/Ca^2+^ pathways in mammals will help understand their role in the pathogenesis of various metabolic derangements. Subsequently, it can provide therapeutic hints for developing personalized medicine against diseases like DM.

Garlic (*Allium sativum L.*) is a globally consumed spice and contains a broad spectrum of bioactive compounds ranging from allicin to S-allyl-cysteine [23]. Significant research has demonstrated that these bioactive components display antioxidant, anti-inflammatory, antibacterial, immunomodulatory, cardiovascular-protective, anticancer, anti-diabetic, anti-obesity, neuroprotective, and renal-protective properties [24, 25]. Bioactive compounds present in garlic serve as an outstanding natural reservoir of pharmaceutically active compounds, holding significant potential for integration into the formulation of functional foods or nutraceuticals aimed at averting and controlling specific diseases [26]. Garlic has diminished pancreatic cell injury, oxidative stress, and pathological alterations in DM [27]. A meta-analysis involving 768 individuals diagnosed with type 2 diabetes mellitus across nine randomized controlled trials revealed a notable reduction in fructosamine and glycosylated hemoglobin levels with garlic supplements [28].

Although various previous studies have reported the pathogenic role of NF-κβ/ TLR-4 and SERCA/Ca2 ^+^ pathways and, subsequently, clinical and therapeutic benefits of AGE against DM, there is an urgent need to identify the potential hotspot targeted by therapeutically active compounds present in AGE[29, 30]. Preliminary findings indicate that dysregulation of NF-κβ/ TLR-4 and SERCA/ Ca2 ^+^ pathways are involved in the pathogenesis of T2DM, and these pathogenic hotspots can be effectively targeted by therapeutically active compounds present in AGE [29, 31, 32]. Henceforth, In the current study, we intend to evaluate the role of NF-κβ/ TLR-4 and SERCA/Ca2 ^+ ^pathways in the pathogenesis of T2DM and its modulation by ethanolic extract of Aged Garlic Extract (AGE).

## Materials and methods

### Chemicals, reagents, and kits

Citrate acid buffer, Streptozotocin, bovine serum albumin, sodium azide, 0.1 M sodium phosphate buffer, 1-deoxy-1-morpholino-D-fructose, glucose, insulin, RPMI-1640 culture media, 10% Fetal bovine serum,100μg/ml streptomycin, ATP assay kit, 2µM Fura-2 AM, pluronic F-127, trypsin-EDTA, Annexin V, 7-AAD, RNeasy Mini Kit, SYBR Premix Ex Taq Kit, hematoxylin and eosin, Picro-Sirius red stain, 2.5% glutaraldehyde solution.

### Animal model

The current study employed adult Wistar rats of both genders weighing between 150 and 180 g. These animals were housed in clean polypropylene cages within a controlled animal facility, maintaining standard temperature and humidity conditions. All protocols involving the utilization of experimental animals adhered strictly to the directives outlined by the Institutional Animal Ethics Committee (IAEC) under Approval No.: Au/FVS/PS-57/9713, which the Committee duly accredits for Control and Supervision of Experiments on Animals (CPCSEA), located in New Delhi, India.

### Preparation and characterization of AGE

AGE was prepared as per the method of Wang et al*.,* 2015 [33] and characterized by gas chromatography-mass spectrometry (GC-MS) and SEM (Scanning Electron microscope) as by Ashraf et al*.,* 2017 and Elosta et al*.,* 2017 [34, 35], respectively. Briefly, GC-MS was conducted by Utilizing narrow capillary columns. The sample was subjected to a temperature of 250 °C at the detection point and 220 °C upon injection, employing helium gas as the carrier at a 1 mL/min flow rate. The analyte (1 µg of advanced glycation end products) was dissolved in triple distilled water at 50 °C, gradually increasing to 280 °C within 5 min. The AGE sample underwent a 30 min run, resulting in the acquisition of a chromatogram. Biologically potent elements were discerned by cross-referencing their relative retention times (RT) with established standards from the National Institute of Standards and Technology (NIST) library database. In the context of SEM, a finely ground composite of advanced glycation end products (AGEs) underwent digestion in laboratory-grade nitric acid within a sealed vessel microwave apparatus (CEM, Germany) operating at 160 °C. Subsequently, the filtrate obtained was analyzed via inductively coupled plasma mass spectrometry (ICP-MS) using helium mode.

#### Estimation of ED_50_

In this current investigation, the moving average method, as outlined by Thompson 1947 [36] was employed to derive generalized equations for estimating the logarithm of the ID_50_. The following prerequisites were followed for the calculation of ED_50_.

i. At every dosage level, a consistent number of animals was employed.
ii. The dosage levels increased exponentially within each tier with a geometric factor (R) 2.0.
iii. The number of animals in each dosage level was equivalent to K + 1, where K represents the number of doses administered.

The equation used to compute the effective dose for 50% of the subjects was

Log m = Log Da + d × (f + 1).

Where ‘d’ represents the logarithm of the constant ratio among dosage levels. ‘Log Da’ signifies the logarithm of the lowest of the four dosage levels used, and ‘f’ denotes the degrees of freedom.

In the current study, we calculated ED_50_ of AGE based on twin critical responses of death and amelioration of hyperglycemia in diabetic animals. Diabetes was induced in laboratory rats as per Elkhalifa et al., 2024 [37]. Briefly, laboratory animals were treated with an intraperitoneal injection of streptozotocin (STZ) at a 45 mg/kg dose dissolved in Citrate acid buffer (CAB). Following intraperitoneal injection of STZ, laboratory animals were screened for induction of diabetes based on blood glucose levels above threshold levels of 280 mg/dl as per the established criteria [38]. As per [38], the requirements for evaluating successful induction of diabetes include persistent hyperglycemia on three estimations, polyphagia, polydipsia, and polyuria observed on the 3^rd^ day, 5^th^ day, and 7^th^ after intraperitoneal injection of STZ. Following this, we conducted a toxicity trial on diabetic laboratory rats. These rats were orally exposed to varying concentrations of AGE, with Group I receiving 125 mg/kg body weight of AGE, Group II receiving 250 mg/kg body weight of AGE, Group III receiving 500 mg/kg body weight of AGE, and Group IV receiving 1000 mg/kg body weight of AGE, administered through oral gavaging for seven days. Each group consisted of 10 animals. After seven days of treatment, initiation of AGE, mortality pattern, and amelioration of hyperglycemia in diabetic animals were recorded, and ED_50_ was calculated.

### Experimental design

Seven days before the experiment, Wistar rats were given time to adjust in the laboratory animal facility, adhering to standardized environmental conditions (22–25 ℃and a 12 h light/dark cycle). After an acclimatization period of 7 days, laboratory animals were randomly divided into four groups. (i) Baseline control group (n = 6): animals in this group were injected with citric acid buffer (CAB) followed by oral administration of phosphate buffer saline (PBS) after 72 h of CAB injection. This group was used to evaluate baseline parameters for comparison with other groups. (ii) AGE-control group (n = 6): animals in this group received AGE at a dose of 250 mg/kg orally suspended in PBS for eight weeks. In the remaining animals (n = 28), DM was induced per the standard procedure of [37]. Briefly after acclimatization, on the eighth day, the animals in this group underwent an overnight fast, followed by the induction of diabetes on the morning of the ninth day through a single intraperitoneal injection of streptozotocin (STZ) at a dose of 45 mg/kg dissolved in CAB with a pH of 7.2. These animals were fed ad libitum and blood glucose level was measured after 72 h of STZ administration for screening of successful DM induction hyperglycemia (blood glucose levels ≤ 280 mg/dl), polydipsia, polyuria, and polyphagia. Animals in which DM was sucessfully induced were included in the current experimental design and divided into two groups (iii) diabetic control group (n = 14) treated with PBS for eight weeks and (iv) AGE treatment group (n = 14) treated with AGE at a dose of 250 mg/kg body weight for eight weeks. At the end of the experiment, blood samples and tissue samples were collected from animals of each group under general anesthesia (thiopental sodium, intraperitoneally at a dose of 50 mg/kg), and samples were processed for biochemical and other laboratory analysis.

### In-vitro anti-glycemic and antioxidant assay

In-vitro protein glycation assay was performed as per Wang et al*.,* 2015 [33], briefly 10% of bovine serum albumin was incubated in the presence and absence of AGE for 24 h, and protein glycation assay was conducted as per the standard procedure. Interrupted post-Amadori assay was performed as per Elosta et al*.,* 2017 [35]. Briefly, the Amador mixture (0.5 M ribose, three mM sodium azide, and bovine serum albumin (BSA) at a dose of 10 mg/ml in 0.1 M sodium phosphate buffer) was incubated at 37 ℃ for 24 h. Subsequently, we assessed the Advanced Glycation End Products (AGEP) levels in the Amodor mixture. Similarly, we conducted a fructosamine assay as per Mahajan et al*.,* 2018 [39] by a calorimetric method using 1-deoxy-1-morpholino-D-fructose (DMF) as standard. In-vitro antioxidant activity of AGE was determined based on free radical scavenging activity based on ABTS and DPPH assay as per Re et al*.,* 1999 and Katsube et al*.,* 2004 [40, 41], respectively.

### Metabolic studies

The intraperitoneal glucose tolerance test (IPGTT) was conducted by keeping laboratory rats fasting for 24 h [42]. After fasting, each rat was given an intraperitoneal injection of glucose at a dose of 2 g/kg body weight. Blood glucose concentrations were assessed at intervals of 0, 15, 30, 60, and 120 min following intraperitoneal glucose administration. In a parallel manner, an intraperitoneal insulin tolerance test (ITT) was administered to laboratory rats following a 6 h fasting period. Subsequently, insulin was intraperitoneally injected at 0.75 U/kg based on the rats’ body weight. This was followed by another intraperitoneal injection of glucose at a dosage of 2 g/kg of body weight. Serum insulin levels were assessed at 0, 15, 30, 60, and 120 min post insulin injection.[9]. The cumulative area beneath the curves (AUC) following glucose or insulin administration was computed using GraphPad Prism version 5.00 for Windows, developed by GraphPad Software in San Diego, California, USA.

### Isolation of β cells of the pancreas

Β-cells of the pancreas were isolated as per Zhang et al*.,* 2020 [43]. A briefly intact pancreas was carefully isolated and typed. A solution containing XI collagenase enzyme was introduced into the pancreas via the common bile duct and placed at room temperature for 17 min. The digested organ homogenate obtained was subjected to dense gradient centrifugation to separate exocrine and endocrine cells. The β-cells isolated from the pancreas were cultured in RPMI-1640 culture media and were stored in RPMI-1640 media supplemented with 10% Fetal bovine serum (FBS) and 100 μg/ml streptomycin.

### Measurement of insulin secretion efficacy and ATP content

The isolated β-cells of the pancreas were incubated in Krebs Ringer bicarbonate buffer supplemented with different concentrations of glucose solution (3.3 mM, 8.3 mM, 11.1 mM, and 16.7 mM) for approximately 30 min [44]. After incubation for 30 min, the supernatant was collected for insulin secretion assay (Invitrogen; Massachusetts, USA; Cat #BMS2003) as per the method of [45]. To quantify the ATP levels within the pancreas, pancreatic β cells underwent lysis, and the ATP content was assessed using an ATP assay kit (Thermofisher; Massachusetts, USA; Cat #A22066) as per method of [46]. Subsequently, the ATP content was adjusted relative to the protein concentration of the supernatant. [44, 47].

### Intracellular calcium content measurement

The calcium levels within the cytosol were quantified using the ratio metric calcium indicator Fura-2 AM (Sigma) [48]. Briefly, cells were cultured at 37 °C in phosphate buffer containing 2.8 mM glucose 2 µM Fura-2 AM and pluronic F-127 for one hour. After an incubation period of one hour, the cells underwent rinsing with distilled water, followed by examination using a fluorescent microscope, utilizing excitation wavelengths of 340 and 380 nm. The 340/380 fluorescence emission change was measured and analyzed under Metafluor imaging software. The fluorescence of Fura-2 AM was considered as baseline/control fluorescence and indicated as F0. In contrast, FΔ was suggested as a change in fluorescence, and cytosolic calcium levels were measured as the ratio of FΔ and F0 [49].

### Apoptosis assay

Apoptosis assay was conducted per the standard procedure of CCK-8 apoptosis assay as per Mitchell et al*.,* 2003 and Beger et al*.,* 2008 [50, 51]. The mentioned protocol involved the dispersion of isolated β cells using trypsin-EDTA, which were then spread onto nitroglycerine-coated glass plates. Subsequently, they were stained with Annexin V and 7-AAD and subjected to analysis using a BD LSRFortessa flow cytometer. β-cell apoptosis was assessed by quantifying the ratio of Annexin V-positive cells using color gradient analysis, with cells classified into early and late apoptotic categories. Furthermore, Caspase-Glo 8 Assay (Promega) and Caspase Glo 3/7 Assay (Promega) were conducted in β-cells of the pancreas. Briefly, β-cells of the pancreas were incubated with Caspase-Glo reagent for one hour, followed by fluorescence measurement under a fluorescent microscope.

#### Western blot and immunoprecipitation

Following established protocols, pancreatic β-cells were harvested and underwent cellular lysis in RIPA buffer supplemented with protease and phosphatase inhibitors. Protein concentration was determined as per Mitchell et al*.,* 2003 [50], followed by the transfer of 30 µg protein to 4–12% NuPAGE gel and its transfer to PVDF membranes. These membranes were incubated for 12 h with various primary antibodies at concentrations of 1: 1000 dilution, which includes anti-APACHE-II, anti-cleaved caspase-8, anti-caspase-8, anti-cleaved caspase-3, anti-FADD, anti-TLR-4, anti-P-62 and anti-NF-kβ. After primary antibody treatment, the membranes were incubated with secondary antibodies for 1 h at 37 °C. Visualization of membranes treated with secondary antibodies was performed utilizing a Bio-Rad imaging system.

#### RNA isolation and quantitative PCR

The quantity of RNA (NF-κβ/ TLR-4 and SERCA/ Ca^2+^) extracted from Pancreatic β-cells was determined following the protocol outlined in the RNeasy Mini Kit (Qiagen, Hilden, Germany). Quantitative PCR in real-time was performed utilizing the SYBR Premix Ex Taq Kit, with subsequent normalization of outcomes relative to the mRNA levels of β actin [37, 52].

#### Microscopic examination

Tissues underwent standard histopathological processing procedures, with 5 µm tissue samples prepared for hematoxylin and eosin (H&E) staining to assess fibrosis. Pancreatic samples were specifically stained using Picro-Sirius red stain (PSR) for analysis, as per Suvarna et al*.,* 2018 [53]. The histopathological changes were scored by Meir et al*.,* 2005 [54]. Briefly, the ratio of β-cell area was counted manually and expressed as the total pancreatic area ratio. Similarly, the percentage of insulin-positive β cells was counted manually in 100 fields per specimen. For evaluation of β-cell apoptosis and β-cell regeneration, TUNEL staining was conducted to identify breaks in DNA strands; in addition to TUNEL staining, cleaved caspase-3 assay (which represents the final step in the apoptosis pathway) was undertaken to serve as an additional marker for detection of β-cell apoptosis. To evaluate pancreatic β-cell regeneration, Ki67 assay in paraffin-embedded pancreatic tissue samples was conducted to enumerate Ki67 positive dividing β cells, which serves as an indirect marker for pancreatic β-cell regeneration.

For SEM examination, pancreatic specimens were preserved in a 2.5% glutaraldehyde solution for 48 h. After 48 h, specimens were sliced into 1 mm thickness and dried with graded alcohol solutions (70%, 80%, 90% and 100%). The dried samples were coated with gold sputtering (20 mm thickness), and gold samples were observed under SEM (SEM-JEOL 100CX II-ASID 4D, Tokyo, Japan) under a voltage of 10–15 kV. SEM images of four experimental groups were compared for their typical and differentiating characteristics.

#### Statistical analysis

Normality testing was performed for each dataset, followed by analysis tailored to the distribution characteristics of the data [55]. Data exhibiting a normal distribution underwent analysis using unpaired Student’s two-tailed t-tests for comparisons between two groups and ANOVA with Tukey’s post hoc test for comparisons involving more than two groups within the study. Likewise, the Mann–Whitney U test (for two groups) or the Kruskal–Wallis test, followed by Dunn's post hoc analysis (for more than two groups) can be employed for analyzing non-parametric datasets in scientific and medical contexts. Statistical analysis was performed using SPSS (version 23), and the data were presented as mean ± Standard Error (SE). Results with a significance level of P ≤ 0.05 were deemed statistically significant.

## Results

### AGE characterization

**Fig. 1:**
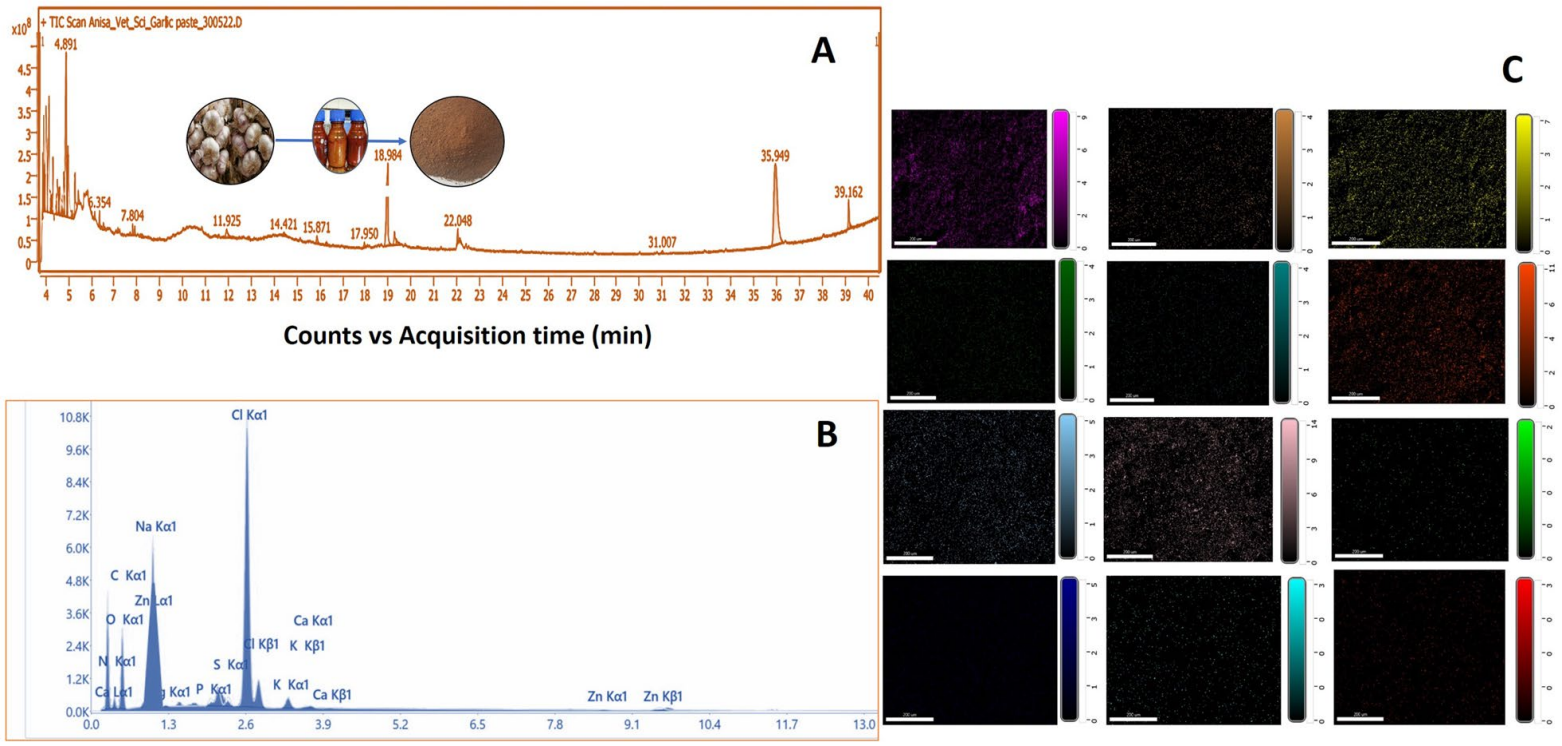
**A** GCMS characterization of AGE revealed the presence of characteristic peaks corresponding to the presence of pharmacologically active ingredients corresponding to phenylalanine, alliin, S-allylmercaptocysteine (SAMC), tryptophan, 1-methyl-1,2,3,4-tetrahydro-β-carboline-3-carboxylic acid, n-Hexadecanoic acid, Methyl 8,9-Octadecadidienoate, tris (Tert butyl dimethyl siloxy) arsane, methyl allicin, tris (butyl dimethyl siloxy) arsane and γ-glutamyl-S-alk(en) L cysteine. **B, C** Elemental profiling of AGE, which gives the chemical and elemental constitution of AGE corresponding to presence of Carbon, Nitrogen, Oxygen, Copper, Zinc, and Selenium as the prominent elements present in AGE

GC-MS analysis revealed 41 significant constituents in AGE, with prominent constituents belonging to phenols, ketones, aldehydes, and phenolic entities. The important entities with maximum peak values were at retention time (RT) of 4.891, 6.354, 7.804, 11.92, 15.87, 17.95, 18.98, 22.04, 31.00, 35.94 and 39.16, and molecular entities identified at these RT values were phenylalanine, alliin, S-allylmercaptocysteine (SAMC), tryptophan, 1-methyl-1,2,3,4-tetrahydro-β-carboline-3-carboxylic acid, n-Hexadecanoic acid, Methyl 8,9-Octadecadidienoate, tris (Tert butyl dimethyl siloxy) arsane, methyl allicin, tris (butyl dimethyl siloxy) arsane and γ-glutamyl-S-alk(en) L cysteine, respectively (Fig. 1A).

**Fig. 2:**
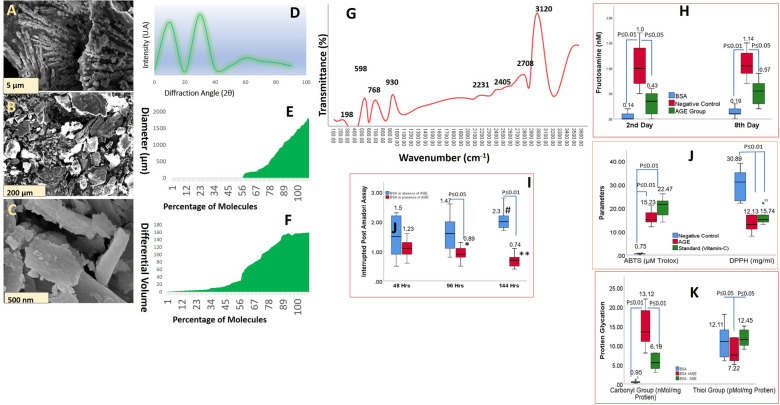
**A–C** SEM micrographs of AGE obtained by lyophilization process at magnification of 5 µm, 200 µm, and 500 nm. **D** X-ray diffractogram of AGE, which indicates the presence of two broad peaks at 2θ of 18.2° and 29.5°, indicates an amorphous structure with minimal crystallinity. **E** Particle size distribution of AGE ranges from 1324 µm to 2314 µm with a porosity of 57% with majority of constituent paricle size falling in range of 500 µm to 2000 µm. **F** Differential volume distribution of AGE revelaing more than 78% of molecules having differential volume from 80 to 180. **G** FTIR spectra of AGE with characteristic peaks at 3900 cm^−1^ for OH and NH_2_, 2810 cm^−1^ for CH_X_ groups, 1543 cm^−1^ for CONH_2_ groups, 1210 cm^−1^ for OH groups, and 947 cm^−1^ for C–N bonding. **H** Fructosamine assay of AGE at intervals of the 2nd and 8th day. **I** Interrupted post-amadori assay of AGE, which indicates the inhibitory activity of AGE against the formation of Advanced glycation end products (AGEP). **J** In-vitro antioxidant assay of AGE measured as per DPPH and ABTS protocol, these assays reveals significant inhibition activity by AGE, these assay revealed significant antioxidant activity of AGE as observed based on DPPH and ABTS assay which indicates antioxidant properties of AGE. **K** protein glycation assay of AGE, which measures the formation of glycated products and modification of protein thiol groups. AGE was found to inhibit the formation of modified protein products. The figure illustrates the results of a protein glycation assay focusing on Advanced Glycation End Products (AGEP). This assay was designed to measure the formation of glycated products and the subsequent modification of protein thiol groups, crucial indicators of glycation-associated protein damage

SEM characterization revealed the presence of cuboidal agglomerates and irregular shapes of varying sizes and resembled broken glass or flake-like structures; some particles exhibited smooth texture with cracks, fissures, and pores on the surface. On SEM analysis, particle size ranged from 1324 µm to 2314 µm and porosity of 57% (Fig. 2A–F). The particle size distribution and value of average diameter, D10, D50, and D90, is shown in Fig. 2E; the shape of These agglomerates is proposed to act as a repository of pharmaceutically biological active principles, which release these principles in a sustained manner. X-ray diffraction of the characteristics of AGE revealed the presence of carbon, nitrogen, oxygen, copper, zinc, and selenium, which are the prominent elements present in AGE (Figs. 1B, C). The Zeta potential of AGE was found to be 19.11 ± 3.17 mV, which indicates the presence of a positive charge on the surface of particles; these findings are of interest in the present study as earlier studies have observed that zeta potential below −30mv and above + 30 mv results in stable emulsion due to electrostatic repulsion between constituent particles [56]. Henceforth zeta potential of 19.11 ± 3.17 mV in the current study indicates unstable emulsion, which results in the formation of agglomerates and coacervates.

The moisture content of AGE was found to be 3.09 ± 1.05% with hygroscopicity of 19.09 ± 6.51%. Low moisture content imparts stability during storage, low endogenic and exogenic enzymatic activities, and inhibits the growth of microorganisms. High hygroscopicity indicates the presence of hydrophilic side chains molecular entities of AGE, which absorb water from ambient air. X-ray diffraction (XRD) analysis revealed the presence of two broad peaks at 2θ of 18.2° and 29.5°, which indicates an amorphous structure with minimal crystallinity (Fig. 2D). Fourier Transform Infrared Spectroscopy (FTIR) analysis of AGE revealed characteristic peaks at 3900 cm^−1^ for OH and NH_2_, 2810 cm^−1^ for CH_X_ groups, 1543 cm^−1^ for CONH_2_ groups, 1210 cm^−1^ for OH groups, and 947 cm^−1^ for C–N bonding (Fig. 2G).

#### Calculation of ED_50_

The ED_50_ was determined by assessing twin critical responses of mortality rates and amelioration of hyperglycemia in laboratory animals within each experimental group over a 7 day longitudinal trial. At the terminal point of the toxicity trail, all animal groups exhibited blood glucose levels below the threshold of 250 mg/kg b.w. The mortality pattern observed across the different groups was 2 and 3 mortalities in the 125 g/kg weight group and 250 mg/kg body weight group, respectively. 9 mortalities were observed in the 500 mg/kg body weight and 10 in the 1000 mg/kg body weight group. All these solutions were dissolved in 10 ml of distilled water for gavaging.

So.

Log m=Log Da + d × (f + 1).

Log m=log [57] + 0.30 (0.125 + 1). The value of f is calculated from values corresponding to 2,3,9 and 10 as mortality pattern, n=10 and K=3.

Log [m]=2.09+0.30(0.125+1)

Log [m]=2.4275

Henceforth m[ED_50_]10ml=2528 mg

So, adjusting for dilution factor ED_50_=252.8 mg.

### In-vitro antioxidant and anti-glycemic activity of AGE

The in-vitro antioxidant assay revealed significant antioxidant activity of AGE, as observed in the DPPH and ABTS assays (Fig. 2J). Results of these assays showed that AGE exhibited antioxidant activity comparable with the free radical scavenging activity of ascorbic acid. Similarly, in the current study, fructosamine and protein glycation assay revealed a significant decline in levels of fructosamine and protein glycation in the presence of AGE compared to levels of protein glycation and fructosamine in the absence of AGE (Fig. 2H, I). Similarly, we found a significant decline in the formation of AGEP on the 8th day of incubation of sugar-free filtrate in the presence of AGE (Fig. 2K).

### AGE improves glucose homeostasis by acting on pancreatic β-ell without improving peripheral sensitivity

**Fig. 3:**
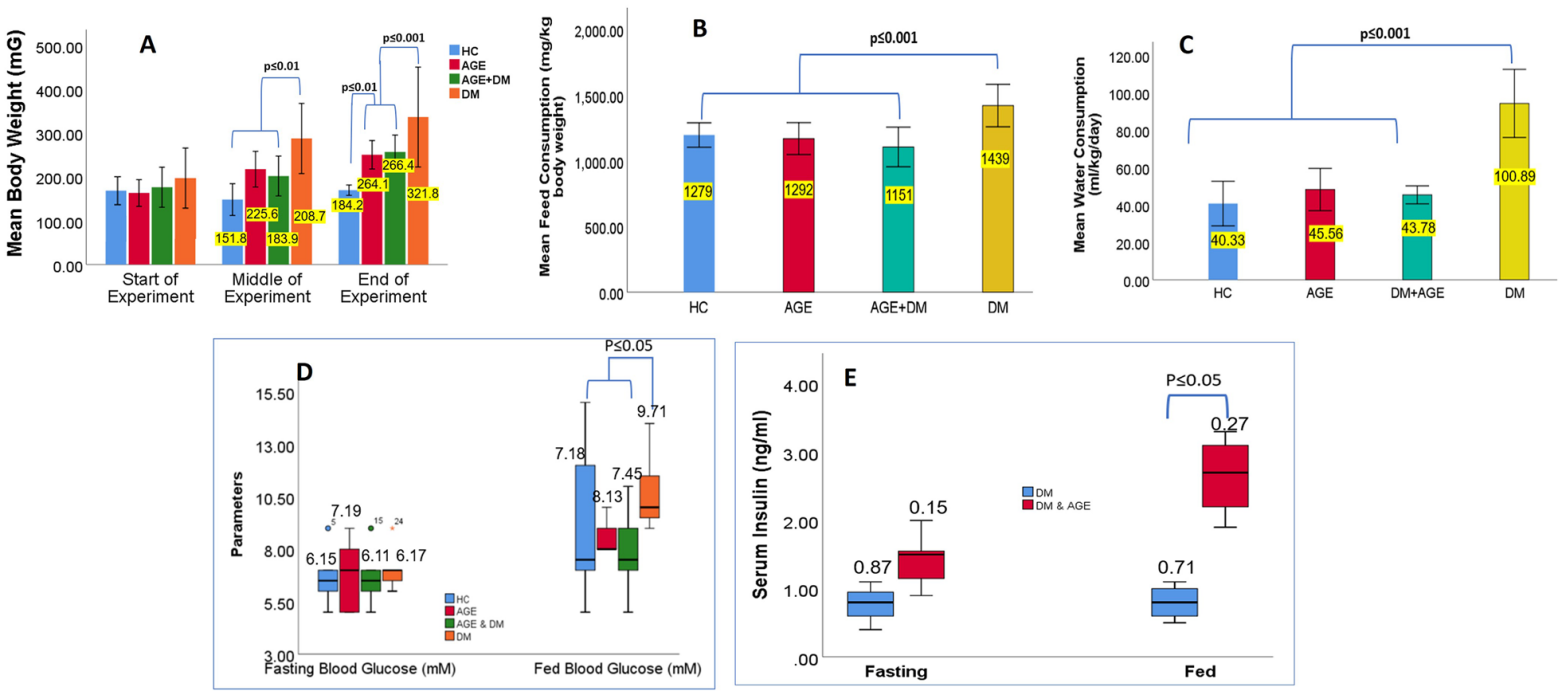
**A** Changes in Body Weight of Laboratory Animals Across Different Time Intervals of Experiment. This graph illustrates the dynamic alterations in the body weight of laboratory animals over the course of the experiment, spanning form start of experiment to end of the experiment. Each data point represents the mean body weight ± standard error of the mean of animals within the specified time interval. **B** Distinctive data series corresponding to different experimental groups or conditions under investigation. Specifically, the figure distinguishes between diabetic animals receiving Aged Garlic Extract supplementation and those subjected to control conditions, enabling direct comparisons of their feed consumption dynamics. **C** Figure illustrates the water consumption patterns observed in laboratory animals, The experiment encompasses diabetic animals subjected to supplementation with Aged Garlic Extract (AGE). Each bar represents the average water intake measured across a specific time period, with error bars indicating the standard deviation. **D** Fasting blood glucose levels of various experimental groups after an overnight fast, juxtaposed with the fed blood glucose levels in diabetic animals with and without supplementation of Aged Garlic Extract (AGE). Each group represents a distinct treatment condition, highlighting the potential impact of AGE supplementation on blood glucose regulation. **E** Figure illustrates the variations in serum insulin levels observed among distinct experimental groups subjected to overnight fasting, alongside random insulin levels detected in diabetic animal models after feeding

There was no significant difference in body weight, water intake, and food intake between the AGE supplementation group and the DM control group (Fig. 3A, B, C). DM was associated with increased body weight gain, and supplementation of AGE resulted in the normalization of body weight in DM animals. This comprehensive analysis of body weight dynamics offers valuable insights into the potential therapeutic benefits of Aged Garlic Extract in managing weight fluctuations associated with diabetes. Likewise, we observed significantly higher food intake (polyphagia) and water intake (polydipsia) in DM group compared to the HC group. Similarly, AGE supplementation results in normalizing food and water intake towards normalcy. The current study observed no significant difference between fasting glucose and insulin levels between the AGE supplementation and DM control groups. Furthermore, we observed a dramatic increase in blood insulin levels after glucose challenge in the AGE supplementation group. However, the AGE supplementation group exhibited significantly reduced random-fed blood glucose levels and concurrently significant elevation in serum insulin levels compared to the DM control group (Fig. 3D, E).

**Fig. 4:**
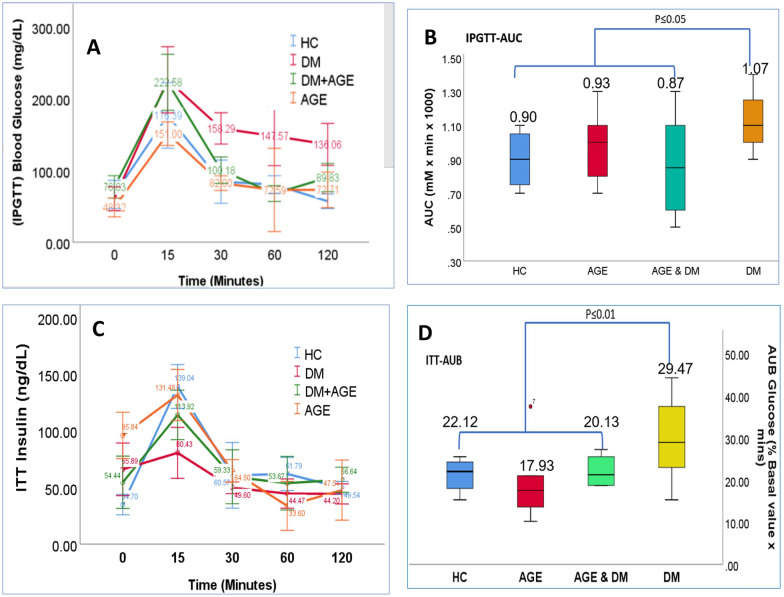
**A:** Comparative Analysis of Blood Glucose Levels among Treatment Groups during IPGTT Intraperitoneal Glucose Tolerance Test (IPGTT) revealed significantly reduced blood glucose values in treatment groups compared to the DM group across different time intervals considered in the present study. Notable reductions in fasting blood glucose levels and enhancements in glucose tolerance were observed upon administration of advanced glycation end products (AGE). **B:** Area Under the Curve (AUC) of Intraperitoneal Glucose Tolerance Test (IPGTT).The AUC of IPGTT significantly decreased in the AGE supplementation group compared to the diabetic control group (p < 0.05). **C:** Comparison of Insulin Sensitivity Among Treatment Groups Area under the curve (AUC) of the insulin tolerance test (ITT) in the diabetes mellitus (DM) group displayed diminished insulin sensitivity compared to other treatment groups (AGE, HC, and AGE + DM) considered in the present study. Subsequent assessment of serum insulin levels revealed a significant elevation in the DM group, which was notably mitigated by administration of advanced glycation end-products (AGE)

Next, we performed an intraperitoneal glucose tolerance test (IPGTT). The results of IPGTT showed significantly lower glucose levels in the AGE supplementation group compared to the DM control group. Likewise, the intraperitoneal insulin tolerance test (ITT) exhibited significant difference in insulin sensitivity between the AGE supplementation group and the DM control group (Fig. 4A–D). Briefly, in IPGTT, blood glucose levels in DM group were found to be significantly elevated across a longitudinal period ranging from 15 min (307.78) to 120 min (436.36) post administration of loading dose of glucose. Likewise, IPGTT reveled significantly reduced blood glucose values in other treatment groups compared to DM group across different time intervals considered in the present study. Given the notable reduction in fasting blood glucose levels and glucose tolerance enhancement upon AGE administration (Fig. 4A). The AUC of IPGTT significantly decreased in the AGE supplementation group compared to the diabetic control group (p < 0.05). The reduced AUC indicated that animals receiving AGE exhibited improved glucose tolerance (Fig. 4B). Our subsequent inquiry focused on the potential impact of AGE on insulin sensitivity in vivo. DM group displayed diminished insulin sensitivity compared to other treatment groups (AGE, HC and AGE + DM) considered in the present study. Subsequent assessment of serum insulin levels revealed a significant elevation in DM group, which the administration of AGE notably mitigated. These findings are consistent with metabolic profiling observed in various studies that indicate these changes are mediated by improving the functioning of pancreatic β-cells (Fig. 4C). In the present experimental design, diabetic animals supplemented with AGE exhibited a significant reduction in AUC compared to the non-supplemented diabetic group. This suggests that Aged Garlic Extract supplementation benefits blood glucose regulation in diabetic animals when analyzed using an ITT approach (Fig. 4D).

**Fig. 5:**
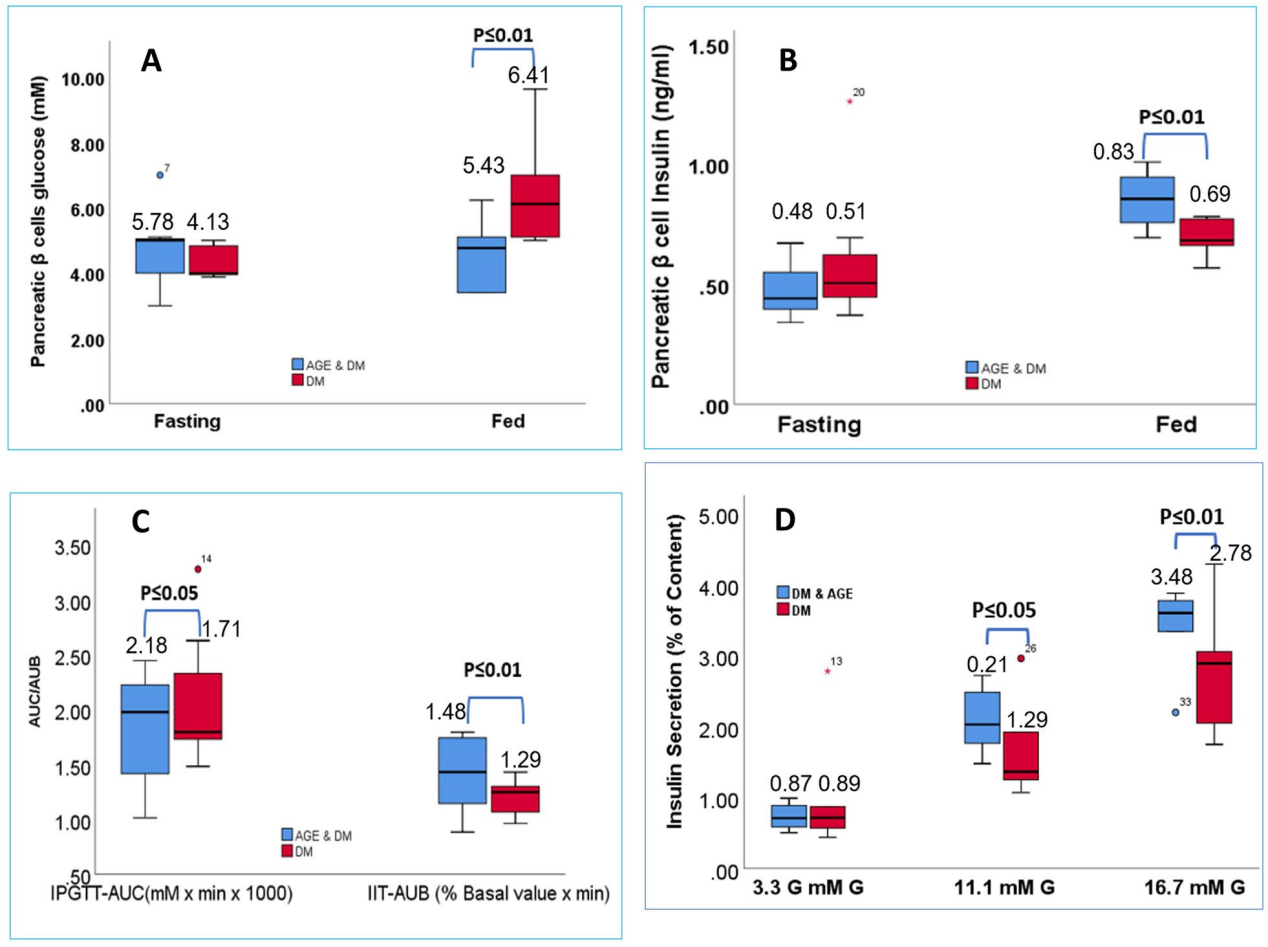
**A:** Effects of AGE Supplementation on Pancreatic Beta Cell Glucose Levels. Under fasting conditions, no significant difference observed between DM groups. In fed conditions, AGE supplementation led to reduced glucose levels in diabetic beta cells compared to controls. No significant differences observed in non-diabetic groups. **B** Fasting: pancreatic beta cell Insulin levels comparable in DM and AGE supplemented DM groups. Fed: AGE supplementation significantly boosts pancreatic beta cell Insulin levels in DM (p < 0.01) compared to untreated diabetes. **C:** Area Under Curve (AUC) of pancreatic beta cell insulin levels and blood glucose levels under fasting and fed conditions in the diabetes group and AGE supplementation group. Insets show the comparative AUC/AUB (Area Under Curve/Area Under Baseline) ratios for insulin and glucose dynamics between the two experimental groups. **D:** Impact of AGE supplementation on pancreatic β-Cell function Pancreatic β-cells isolated from AGE supplementation and DM control groups were subjected to varying glucose concentrations (3.3 mM, 11.1 mM, and 16.7 mM) in vitro. At 3.3 mM glucose, insulin secretion was comparable between groups. However, at 11.1 mM and 16.7 mM glucose challenges, significantly higher insulin levels were observed in the AGE supplementation group compared to the DM control group. Additionally, the stimulation index was significantly elevated in the AGE supplementation group, indicating improved pancreatic β-cell responsiveness to glucose stimulation

Under fasting conditions, no significant difference was observed in pancreatic beta cell glucose levels between DM group and DM group supplemented with AGE. However, AGE supplementation in fed pancreatic beta cell led to a significant reduction in pancreatic beta cell glucose levels in the diabetes group supplemented with AGE compared to the control group. No significant differences were observed in pancreatic beta cell glucose levels between the AGE supplementation and control groups in the non-diabetes group (Fig. 5A). Likewise, Under fasting conditions, no significant difference was observed in pancreatic beta cell Insulin levels between DM and AGE-supplemented DM group. In contrast to this under fed conditions, AGE supplementation demonstrated a notable improvement in pancreatic beta cell insulin levels compared to the untreated diabetes group, with a statistically significant increase observed (p < 0.01) (Fig. 5B). In the diabetes group, fasting insulin AUC was significantly lower than the AGE group (p < 0.05). Fasting blood glucose, AUC was considerably higher in the diabetes group compared to the AGE group (p < 0.01). Under fed conditions, insulin AUC was significantly reduced in the diabetes group compared to the control group (p < 0.05), while blood glucose AUC was significantly higher (p < 0.01). AGE supplementation significantly increased fasting insulin AUC compared to the diabetes group without supplementation (p < 0.01). Fasting blood glucose AUC was considerably lower in the AGE supplementation group compared to the diabetes group (p < 0.05). In the fed state, insulin AUC was significantly higher in the AGE supplementation group compared to the diabetes group (p < 0.01), and blood glucose AUC was substantially lower (p < 0.01) (Fig. 5C). To further assess the role of AGE on pancreatic functioning, the primary pancreatic β-cells isolated from both groups were treated in-vitro with three concentration gradients of glucose (3.3 mM, 11.1 mM, and 16.7 mM). At 3.3 mM glucose concentration, insulin levels secreted by isolated β-cells were comparable. In contrast, significantly higher insulin levels were observed in the AGE supplementation group compared to the DM control group at glucose challenge of 11.1 mM and 16.7 mM, along with significantly elevated stimulation index of the AGE supplementation group (Fig. 5D).

**Fig. 6:**
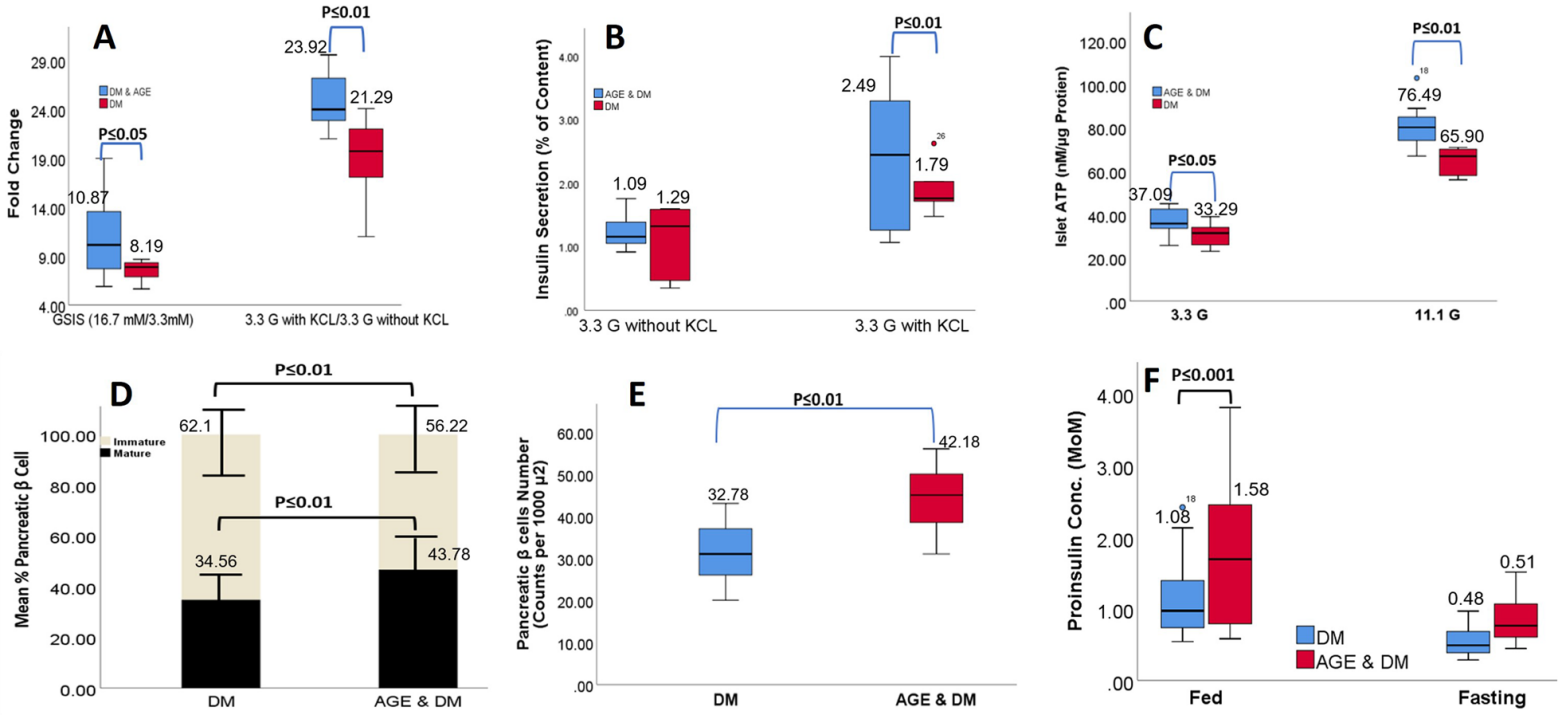
**A:** Figure compares the insulin secretion response to increasing glucose concentrations between the diabetes mellitus (DM) group and AGE supplementation group. The fold increment in insulin secretion levels is depicted over a range of glucose concentrations, highlighting potential differences in insulin secretory capacity and responsiveness between the two experimental conditions. **B:** Evaluation of the role of KCl in modulating insulin secretion in both diabetes mellitus and AGE supplementation groups. Data depict the mean ± SEM of insulin secretion levels in the presence and absence of KCl, emphasizing differential responses between the two experimental conditions. **C**: Islet ATP levels measured under varying concentrations of glucose (3.3 mmol/L). Islets were subjected to different glucose concentrations ranging from basal (2.8 mmol/L) to stimulatory (16.7 mmol/L), and ATP levels were quantified. Each data point represents the mean ± standard error of the mean (SEM) of n independent experiments (n = 6). **D:** Quantitative analysis showing the proportion of immature β cells) relative to total β cells in the DM, AGE, and DM + AGE groups. **E:** Figure illustrates the quantitative assessment of pancreatic β-cell numbers in animal subjects following distinct therapeutic interventions. Each bar represents the mean β-cell count ± standard error of the mean (SEM) across multiple specimens per group. **F:** Schematic representation illustrating the impact of therapeutic intervention on pro-insulin concentrations in pancreatic β cells under different metabolic states (fed vs fasting)

Next, we conducted a KCl-stimulated insulin secretion assay; briefly, isolated β cells were treated with KCl as per the method of [56]; results of this assay revealed significantly elevated insulin secretion in the AGE supplementation group after KCl exposure compared to the DM control group β-cells (Fig. 6A, B). These results indicate that AGE induces insulin secretion by inactivating glucose-induced K_ATP_ channels. These postulates are supported by significantly elevated intracellular ATP levels in AGE supplementation group β-cells (Fig. 6C). To evaluate whether AGE supplementation causes changes in β-cell mass, we stained pancreatic sections for insulin, and our results indicate that ratio of β-cell area/pancreatic area and β-cell mass were significantly elevated in AGE group compared to DM group with no change in pancreatic architecture and no change in the ratio of β-cells/α-cells, contrarily to these findings we could not observe any significant difference in α-cell area/mass between AGE group and DM group (Fig. 6D, E). Consistently we found that pro-insulin (Ins1 and Ins 2) transcriptions were significantly upregulated in the AGE group compared to the DM group (Fig. 6F). These findings collectively indicate that AGE supplementation in DM increases insulin synthesis and secretion from β cells of the pancreas by enhancing structural and functional profiling of pancreatic β-cells.

### AGE causes an increase in β-cell mass by reducing apoptosis of pancreatic β-cell

**Fig. 7:**
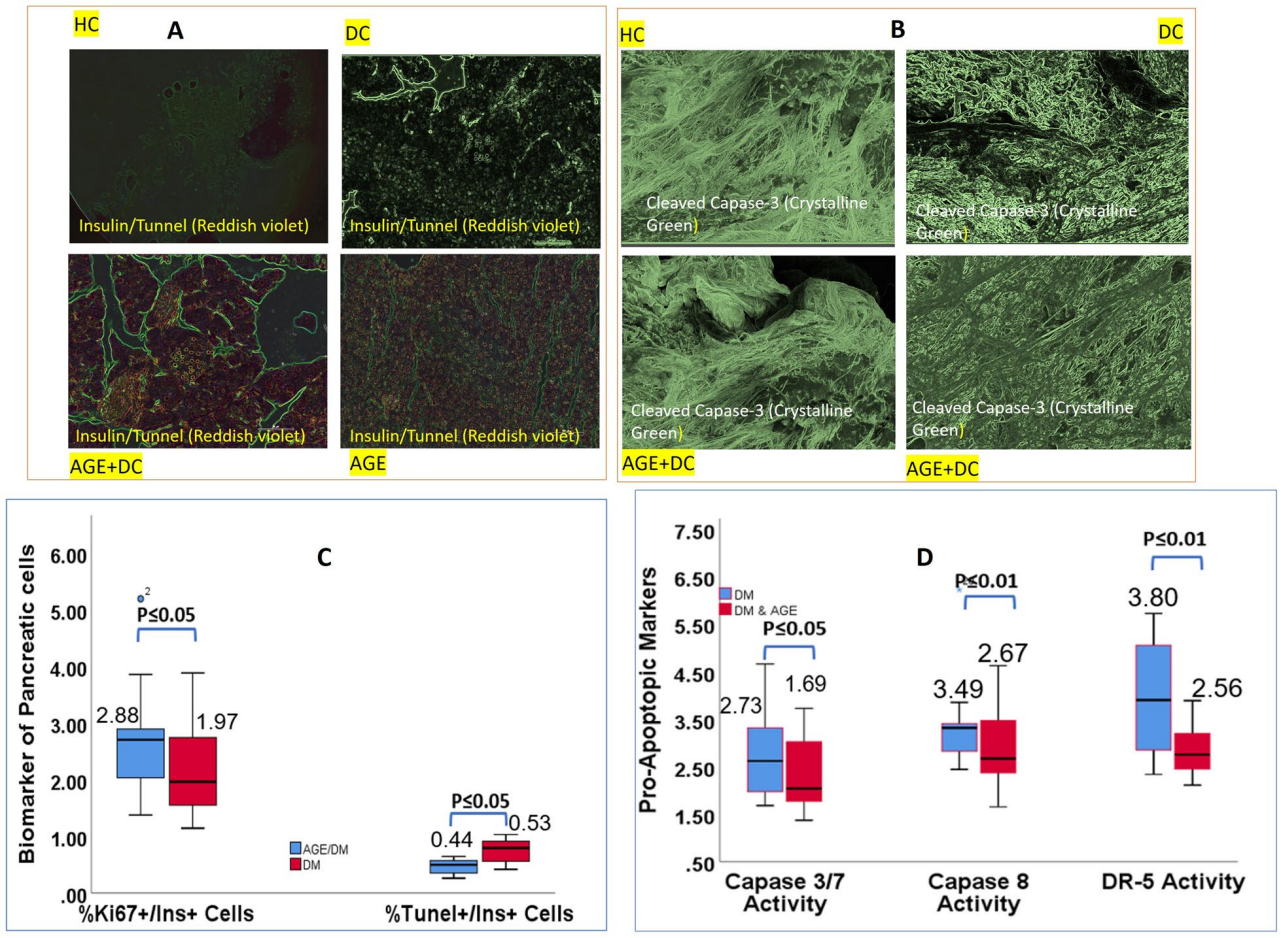
**A** (HC)Representative micrograph showing pancreatic tissue sections from healthy control animals stained with TUNEL assay (green) to detect apoptotic cells. Pancreatic β cells are counterstained with DAPI (blue). Note the minimal presence of apoptotic cells (green) within the pancreatic islets, indicating low levels of apoptosis in healthy control animals. (DC) Representative micrograph illustrating pancreatic tissue sections from disease control animals stained with TUNEL assay (green) to visualize apoptotic cells. Pancreatic β cells are counterstained with DAPI (blue). Increased presence of apoptotic cells (green) within the pancreatic islets is observed compared to healthy controls, indicating elevated levels of apoptosis in disease control animals. (AGE) Representative micrograph showing pancreatic tissue sections from Allergic control animals stained with TUNEL assay (green) to detect apoptotic cells. Pancreatic β cells are counterstained with DAPI (blue). Note the minimal presence of apoptotic cells (green) within the pancreatic islets, indicating low levels of apoptosis in healthy control animals. (AGE + DM) Representative micrograph depicting pancreatic tissue sections from animals supplemented with AGE. Comparatively higher levels of apoptotic cells (green) within the pancreatic islets are observed in the AGE supplementation group compared to healthy controls but potentially lower than in disease controls. **B: **(HC) Pancreatic tissue sections from healthy control animals reveal minimal immunofluorescence signal for cleaved caspase 3, indicating low levels of apoptosis in β-cells under physiological conditions. (DC) In pancreatic tissue sections from animals with the disease condition, a significant increase in immunofluorescence signal for cleaved caspase 3 is observed compared to the healthy control group, indicating elevated apoptosis levels in pancreatic β-cells in the disease state. (AGE + DM)**:** Animals receiving AGE supplementation exhibit a notable decrease in immunofluorescence signal for cleaved caspase 3 compared to the disease control group. This reduction suggests a potential therapeutic effect of the supplemented drug in attenuating apoptosis in pancreatic β-cells. **C:**Quantitative analysis of the ratio of %Ki67 + /Ins + cells and %TUNEL + /Ins + cells in pancreatic islets from experimental groups. Data are presented as mean ± standard error of the mean (SEM) from n independent experiments. Statistical significance was determined by [appropriate statistical test] (*p < 0.05, **p < 0.01, ***p < 0.001 vs. control). **D:** Summary graphs depicting quantitative data obtained from various assays, summarizing the levels of pro-apoptotic markers in pancreatic β-cells across different experimental conditions. Statistical analyses such as performed to determine significant differences (*p < 0.05, **p < 0.01, ***p < 0.001) between experimental groups

**Fig. 8:**
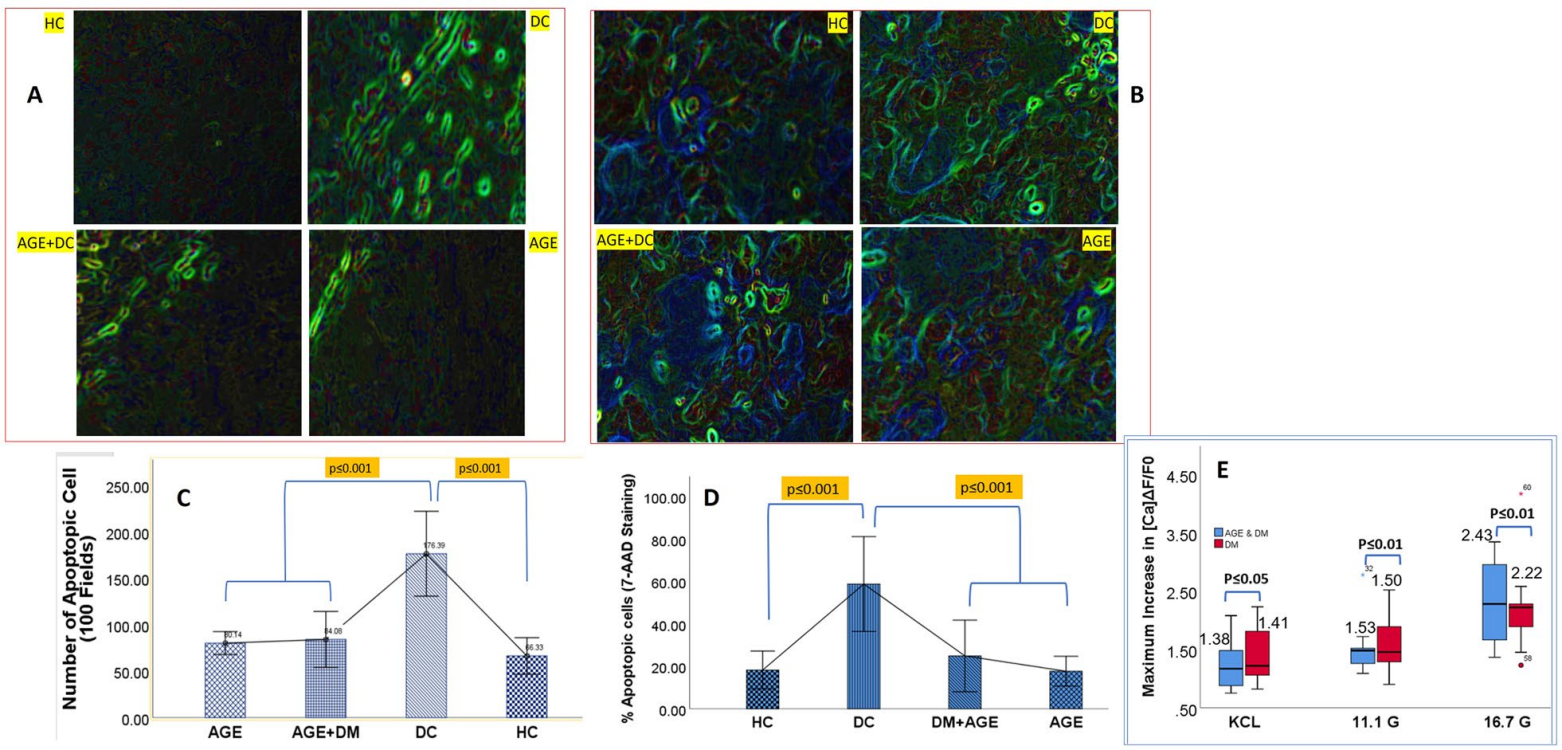
**A:** Pancreatic β-cells of the healthy control group. Minimal Annexin V fluorescence (green) is detected, reflecting low apoptotic activity and maintenance of cellular homeostasis in the healthy pancreatic tissue (HC). Fluorescence micrograph showing Annexin V staining in pancreatic β-cells of the disease control group. Increased Annexin V expression (green fluorescence) indicative of apoptotic activity is observed, signifying cellular stress and dysfunction in the diseased pancreatic microenvironment (DC). pancreatic β-cells of AGE supplementation group. Reduced Annexin V fluorescence (green) is evident, indicative of attenuated apoptotic activity following AGE intervention within the pancreatic microenvironment (AGE + DM). Microscopic visualization illustrating Annexin V staining in pancreatic β-cells of the allergic control group, indicating significantly reduced apoptosis compared to disease control group (AGE). **B** Immunofluorescence co-staining for insulin (green fluorescence) and 7-AAD (red fluorescence) in pancreatic sections from the in different experimental groups indicating compromised β-cell viability in DC group compared to HC group, whileas supplemetation of AGE resulted in inhibition of cellular apoptosis in AGE + DM group. **C:** Comparative Assessment of Annexin-V Positive β-Cells among experimental groups, The analysis provides insights into the impact of AGE supplementation on β-cell apoptosis, comparing it with both pathological and physiological contexts. Data are presented as mean ± standard error of the mean (SEM) from n independent experiments (*p < 0.05, **p < 0.01, ***p < 0.001). **D:** Comparative assessment of the percentage of 7-AAD-positive β-cells among experimental groups. The proportion of 7-AAD-positive cells was calculated from at least ten randomly selected fields per sample and expressed as a percentage of total β-cells. Data are presented as mean ± SEM. **E:** Maximum increment in intracellular pancreatic β-cell Ca2 ^+^ levels in response to stimulation with KCl and varying glucose concentrations (11.1 mM and 16.7 mM)

For evaluation of β-cell apoptosis and β-cell regeneration, TUNEL staining was conducted to identify breaks in DNA strands; in addition to TUNEL staining (Fig. 7A), cleaved caspase-3 assay (which represents the final step in the apoptosis pathway) (Fig. 7B) was conducted to serve as a marker for detection of β-cell apoptosis. To elaborate further on Ki67 and insulin dual-stainings, we could not observe any significant difference in β-cell proliferation, but we observed a substantial reduction in levels of apoptotic Tunel Insulin cells, Death Receptor-5 activity, and cleaved caspase-3 and in AGE-treated β-cell compared to DM β-cell (Fig. 7C, D). Collectively these results indicate pharmacologically active principles of AGE increase the functionality of pancreatic β-cell, which results in incremental changes in insulin levels and inhibits apoptotic pathways and hence maintains the structural integrity of pancreatic β-cells. Furthermore, from Fig. 8A–D, it can be observed that the induction of diabetes mellitus results in elevated apoptotic cell death in pancreatic islets, as indicated by the presence of Annexin V (Fig. 8A, B). Also, AGE supplementation in DM protects pancreatic islet cells against apoptosis. Similarly, 7-AAD staining of pancreatic islets indicated that AGE imparts a therapeutic effect by sparing pancreatic β cells against programmed cell death (Fig. 8C, D).

### AGE causes restoration of calcium (Ca^2+^) stores in β-cells

In the current study, we estimated intracellular calcium levels using the method [54], and we found no significant difference between baseline values of Ca^2+^. Contrarily to these findings, when isolated pancreatic β-cells were subjected to 25 mM glucose solution, pancreatic β cells of the AGE group exhibited a significant increase in Ca^2+^ levels compared to blunted enhancement observed in the DM control group (Fig. 8E). These results are consistent with KCl-activated insulin increment in different treatment groups of the current experimental design.

### AGE mediates insulin secretion through NF-κB /TLR-4 pathway and SERCA/Ca^2+^ pathway

**Fig. 9:**
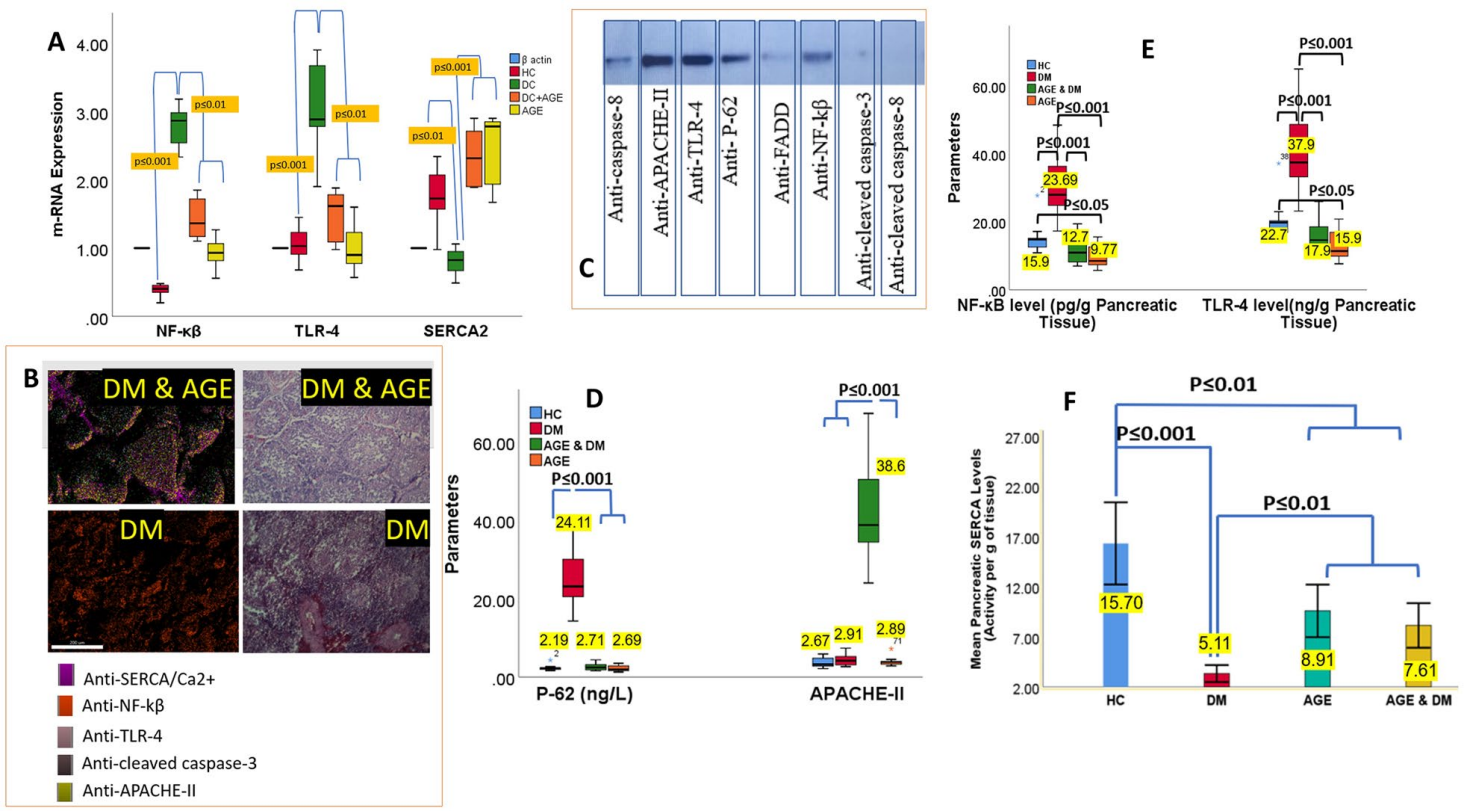
**A:** mRNA Expression Levels of NFκB, TLR-4, and SERCA2 in Different Study Groups. mRNA expression levels of Nuclear Factor Kappa B (NFκB), Toll-like Receptor 4 (TLR-4), and Sarco/Endoplasmic Reticulum Ca2 ^+^ -ATPase 2 (SERCA2) were analyzed in the Diabetes Mellitus Disease Control Group (DM-DC), the Healthy Control Group (HC), and the Aged Garlic Extract Supplementation Group (AGE). mRNA expression levels were quantified using RT-qPCR and normalized to housekeeping genes mRNA levels of β actin. Results depict significant alterations in the expression levels of NFκB, TLR-4, and SERCA2 among the study groups, highlighting potential molecular mechanisms underlying diabetes mellitus and the impact of AGE supplementation. **B-C:** Representative immunohistochemistry micrographs of pancreatic sections derived from diabetic (DM) and diabetic animals supplemented with AGE, stained for Anti-SERCA/Ca2 ^+^ , Anti-NF-kβ, Anti-TLR-4, Anti-cleaved caspase-3, and Anti-APACHE-II. Immunoreactivity against Anti-SERCA/Ca2 ^+^ reveals alterations in calcium handling machinery within pancreatic tissue of diabetic mice compared to controls, suggesting potential dysregulation in intracellular calcium dynamics. Immunostaining for Anti-NF-kβ depicts differential nuclear translocation indicative of inflammatory signaling activation in the diabetic milieu, particularly accentuated in the presence of AGE. Anti-TLR-4 staining highlights enhanced expression of Toll-like receptor 4, implicating its involvement in inflammatory responses characteristic of diabetic pathology, with further augmentation observed in the DM and AGE group. Detection of Anti-cleaved caspase-3 signals apoptotic activity, showcasing increased apoptotic events within pancreatic tissues of diabetic mice, a phenomenon that is exacerbated in the presence of AGE. Immunohistochemical assessment of Anti-APACHE-II demonstrates alterations in pancreatic acinar cell function and integrity, indicative of systemic stress responses associated with diabetic conditions. **D:** P-62 and APACHE-II levels in animals in animals of different experiment groups exposed to therapeutic interventions. **E:** NF-kβ and TLR-4 levels in animals of different experiment groups exposed to therapeutic interventions. **F:** Mean pancreatic SERCA levels in experiment groups exposed to therapeutic interventions

We found an increased mRNA and protein expression in levels of NF-κB and TLR-4 in the DM control group compared to the healthy control group and the AGE control group (Fig. 9A). Furthermore, AGE supplementation in Diabetic animals was found to cause a significant decline in the expression of NF-κB and TLR-4 levels compared to levels observed in the DM control group (Fig. 9A), and NF-κβ/TLR-4 expression and SERCA/Ca^2+^ pathways were evaluated under physiological and pathological diabetic conditions. We found that NF-κβ/ TLR-4 and SERCA/ Ca^2+^ pathways are involved in the mediation of insulin sensitivity, anti-apoptosis of pancreatic β-cells, and recruitment of Ca^2+^, which culminates into the inactivation of the pro-apoptotic pathway. Results of the current study indicate NF-κβ/ TLR-4 and SERCA/Ca^2+^ pathways as potential determinants for the regulation of pancreatic β-cells functioning and, subsequently, tailoring therapeutic targets against T2DM. SERCA/Ca^2+^ pathway has been identified as a predominant pathway for the regulation of β-cell functioning; based on this hypothesis, we conducted Immunofluorescence analyses (Fig. 9B) for SERCA/Ca^2+^, and the results of the current revealed a significant imbalance in SERCA/Ca^2+^ levels with a significantly reduced ratio of SERCA/Ca^2+^ in DM control group. In contrast, the SERCA/Ca^2+^ ratio in the AGE group was close to other healthy groups.

Next, we analyzed the molecular mechanism between these pathways, which involved the evaluation of protein expression of APACHE-II, Caspase-8, TLR-4, P-62, FADD, NF-kβ, cleaved caspase-3 and cleaved caspase-8 which leads to pancreatic β-cell apoptosis (Fig. 9C). Isolated pancreatic β-cells from the DM control group indicated significantly elevated APACHE-II, TLR-4, NF-kβ, and P-62 activity. In contrast, supplementation of AGE caused inhibition in pancreatic β-cells apoptosis pathway and marked by a significant decline in levels of APACHE-II, TLR-4, NF-kβ, and P-62, which indicates causes modulation of apoptosis in pancreatic β-cells (Fig. 9D, E).

To further elucidate the role of Ca^2+^ in the initiation of the apoptosis pathway, we evaluated the concentration of Death Receptor-5 (DR-5) in pancreatic β-cell isolated from different treatment groups, and our studies indicate significantly higher levels of DR-5 in the DM control group compared to other groups of the present study. Henceforth, considering together, it can be postulated that DM involves sequestration of Ca^2+^ inside the Endoplasmic reticulum and is caused by decreased efficacy of SERCA, which initiates activation of NF-κB/TLR-4 apoptosis pathways which subsequently enhances caspase-3/7/DR-5 protein activity in pancreatic β-cells (Fig. 9F).

### Histopathology/microscopic examination

**Fig. 10:**
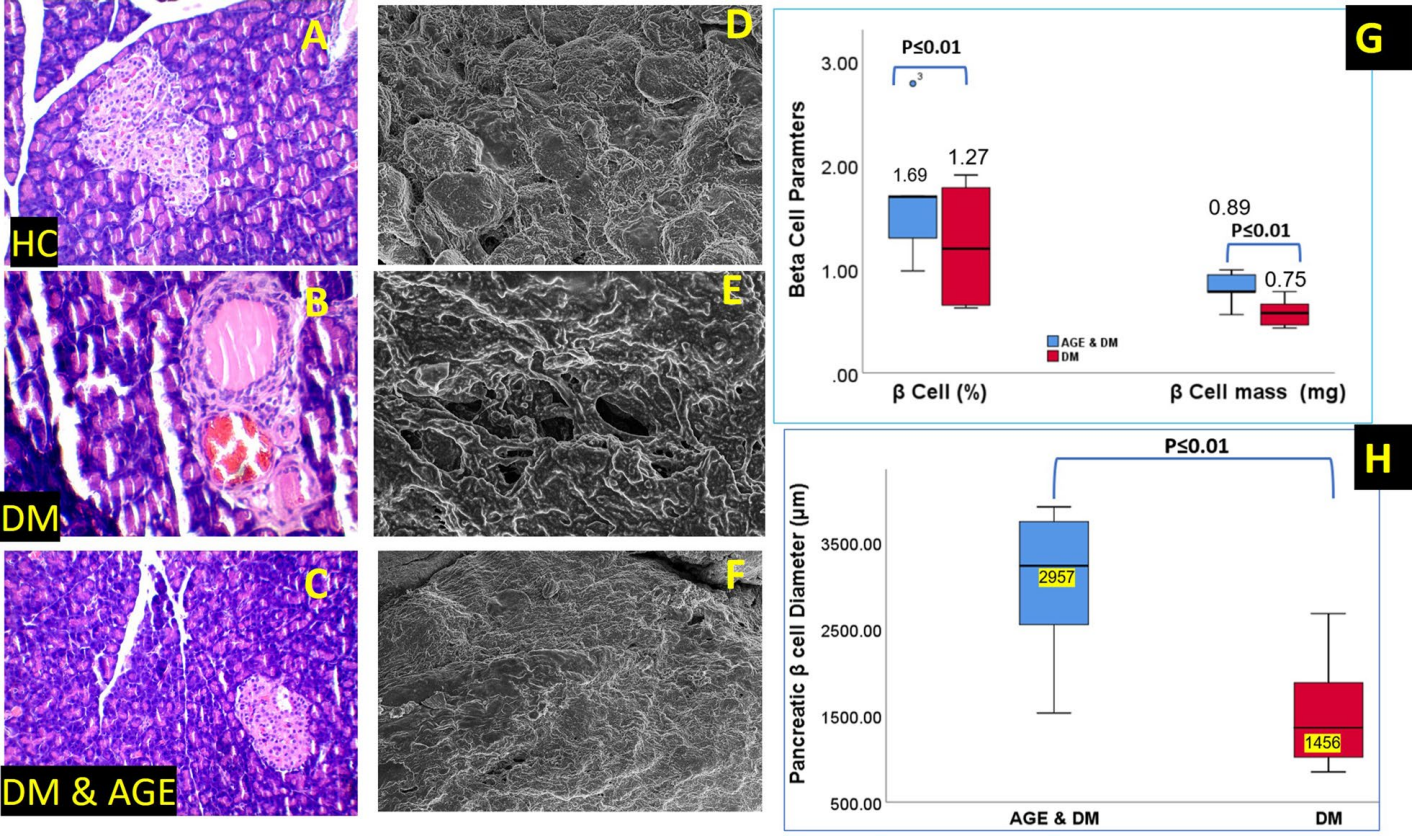
**A–C:** Histopathological changes in the pancreas of different experimental groups reveal normal islets of Langerhans in the healthy group. DM control group exhibited swollen and small acidophilic vacuoles AGE-DM group exhibited less severe apoptotic changes with the intact border between an exocrine and endocrine portion of cells with cellular architecture progressing toward normalcy**. D** SEM of AGE & DM group on 7^th^ day post AGE supplementation. **E:** SEM of AGE & DM group on 14^th^ day post AGE supplementation. **C** SEM of DM group on 7th-day post-induction of Diabetes Mellitus. **F** SEM of DM group on 14^th^ day post-induction of Diabetes Mellitus. **F:** pancreatic β-ell diameter of experiment groups exposed to therapeutic interventions

Histopathological examination of the pancreas in a healthy control group revealed the appearance of normal islets of Langerhans, with lightly stained central islet cells compared to surrounding acinar cells (Fig. 10A). The DM control group exhibited cellular pathological changes with acinar cells being swollen, and small acidophilic vacuoles were observed in almost most cells with complete loss of Islet β-cells of the pancreas (Fig. 10B). Meanwhile, the AGE-DM group exhibited less severe apoptotic changes with intact borders between exocrine and endocrine portions of cells, and their cellular architecture progressed toward normalcy (Fig. 10C). Immunohistochemical staining of pancreatic β cells for anti-insulin antibodies revealed the presence of large granules of insulin staining (Fig. 7A). Similarly, in the AGE-DM group, positive immunoreactions of β-cells for anti-insulin antibodies increased compared to the DM group. The similarly present study reports a significantly higher number of pancreatic β-cells in the HC, AGE, and AGE-DM Groups compared to pancreatic β cells observed in the DM control group. In the HC group, an intact acinar ductal system was observed, while in the DM control group, significantly higher polymorphonuclear lymphocytic infiltration was observed (Fig. 10A–C). On SEM analysis, the normal architecture of individual pancreatic β-cells was observed in the HC group.

**Fig. 11:**
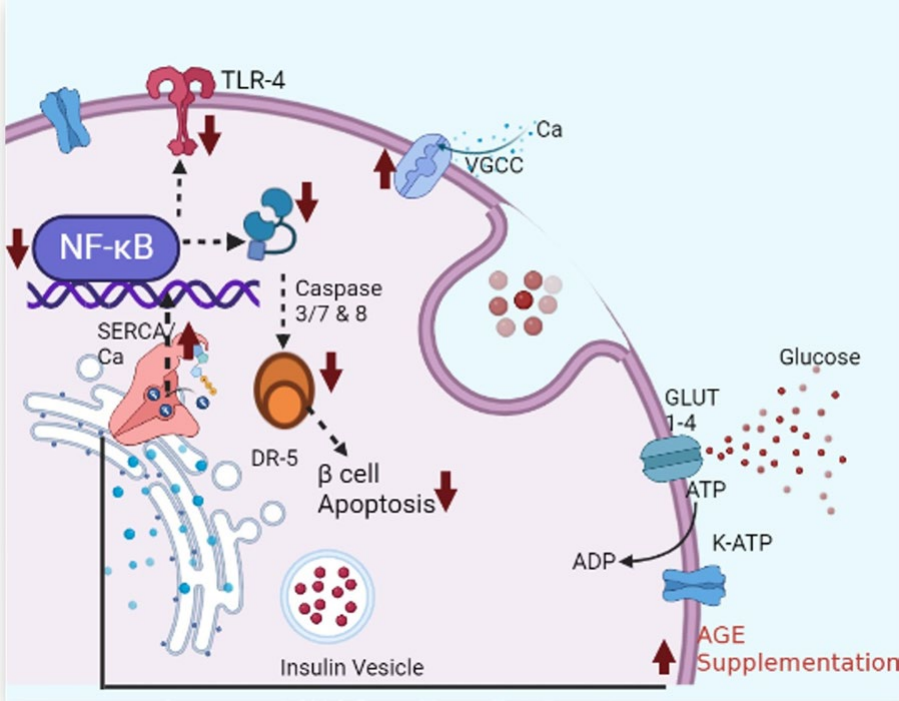
Model depicting the role of NF-κβ/ TLR-4 and SERCA/ Ca^2+^ in the progression of DM. Ca^2^ dysregulation resulting from inhibited SERCA2 activity causes a reduction in secretory functioning of pancreatic β-ell, which reduces blood insulin levels and consequently causes chronic hyperglycemia, which initiates a vicious cycle of pancreatic β-ell apoptosis by activation of DR5/FADD/caspase-8 pro-apoptotic complex which culminates in impaired insulin secretion and aggravated Ca^2+^ induced β-cell apoptosis. AGE exerts its anti-diabetic action by acting on KATP: ATP-sensitive K + channel which causes ingression of glucose into pancreatic β cell, regulating the function of SERCA2 which consequently results in a decline of NF-κβ/ TLR-4 levels and hence inhibits formation of DR5/FADD/caspase-8 pro-apoptotic complex (TLR-4: Toll-Like Receptor-4; VGCC: voltage Gated Calcium Channels; DR-5: death Receptor 5)

In contrast, pancreatic β-cell in DM control exhibited severe fibrosis and calcification with loss of cellular architecture (Fig. 10D, E, F). Β-cell mass and β-cell mass (% pancreas) were significantly (p < 0.001) elevated in HC, AGE, and AGE-DM groups compared to DM control (Fig. 10G, H). Furthermore, results of the current study indicated that levels of autophagic selective substrate P-62 and APACHE-II levels were significantly elevated in the diabetic control group compared to levels of these markers in the healthy control and AGE group (Fig. 9D). In addition, we observed significantly higher levels of Ki-67 positive β-cells in the AGE group compared to the healthy control and disease control groups. These studies indicate the anti-apoptotic activity of pharmacologically active ingredients present in AGE, which protect pancreatic β-cell apoptosis (Fig. 7C). In the current study, we found activation of NF-κβ/TLR-4 and SERCA/Ca^2+^ are involved in the progression of DM. Chronically dysregulation of Ca^2+^ levels in pancreatic β-cells causes an increase in the expression of NF-κβ and TLR-4, and decreased levels of Ca^2^ reduce the secretory function of pancreatic β-cells (Fig. 9E, F). The decline in the secretory capacity of pancreatic β cells causes uncontrolled hyperglycemia, which further aggravates the dysregulation of Ca^2+^ and initiates a vicious cycle of apoptosis by activation of a pro-apoptotic complex of DR5/FADD/caspase-8 which results in the loss of pancreatic β-cell mass and progression of DM (Fig. 7). Furthermore, our results support the beneficial role of AGE on blood glucose homeostasis is mediated by the preservation of pancreatic β-cell mass and pancreatic β-cell functioning by modulating the functioning of NF-κβ/TLR-4 and SERCA/ Ca^2+^ pathways (Figs. 9, 11).

## Discussion

Zeta potential indicates agglomerate formation and aggregation characteristic of lyophilized particles in the dispersion medium. In the current study, zeta potential, 19.11 ± 3.17 mV being below the threshold levels (more than 30 mV or less than 30 mV), results in unstable AGE dispersion formation in the dispersion medium [56, 58]. These findings are in concurrence with SEM findings of agglomerate deposit formation of AGE, as these agglomerates are formed by electrostatic attraction between positive amino groups (NH^3+^) of CH and negative carboxyl groups (–COO−) of various biologically active principles present in AGE [59]. In the current study, the moisture content of AGE was found to be 3.09 ± 1.05% with hygroscopicity of 19.09 ± 6.51%, these values are almost close to the moisture content values of encapsulation of the garlic extract by spray drying process by other authors, and these values indicate good stability characteristics of AGE powder during storage [60]. Furthermore, moisture content below 10% retains functional and structural characteristics of biologically active components intact as moisture content below a threshold level of 10% inhibits hydrolytic reactions, endogenous and exogenous enzymatic activity, growth of microorganisms, and oxidation of functional groups of these components [61]. AGE extract prepared had a hygroscopicity of 19.09 ± 6.51, the hygroscopicity above threshold levels of 5% indicates the presence of hydrophilic side chains available for interaction with biological macromolecules and henceforth exerts their biological action [62].

Furthermore, higher hygroscopicity ensures the diffusion of biologically active components across the cell membrane and imparts its therapeutic activity at cellular and subcellular levels [63]. On SEM analysis, AGE’s particle size ranged from 1324 µm to 2314 µm with a porosity of 57%. In the current study, particle size was more significant than the size reported earlier. Earlier studies have reported AGE particle size ranging from 119.9 to 567.90 µm; this difference might be attributed to process characteristics. Although particle size varies significantly from reports of other authors, particle morphology was almost similar to reports of Burgess [64].

X-ray diffraction of the characteristics of AGE revealed the presence of carbon, nitrogen, oxygen, copper, zinc, and selenium, which are the prominent elements present in AGE. Furthermore, XRD analysis revealed broad peaks at 2θ of 18.2° and 29.5°, indicating an amorphous structure with minimal crystallinity. These findings agree with [65]; they postulated that a diffractogram with two broad peaks indicates crystalline structures distorted by electrostatic attraction, resulting in small amorphous structures within the crystalline mass. Furthermore, similar structural characteristics have been reported by [66], who prepared curcumin extract through lyophilization. The potential advantage of these amorphous extracts for managing DM includes increased bio-availability, sustained release of biologically active compounds, and flexibility in the formulation of drugs and dosages.

Furthermore, amorphous preparations offer the advantage of combining multiple drugs in a single formulation. FTIR spectra bands of AGE in the present study concur with the findings of earlier studies for other related extracts and chitosan [64, 65]. A similar band profile with almost similar wavenumbers of garlic has been reported by other authors [67–69], which indicates the presence of intact biologically active components and, subsequently, retention of their therapeutic efficacy and biological activity. In addition, the absence of any additional/new peak compared to the standard spectrogram reported by other authors indicates the absence of chemical reaction between functional groups of biologically active components present in AGE [70].

In-vitro antiglycation activity of AGE is in agreement with the findings of [71]; the antiglycation action of AGE has been attributed to biologically active compounds present in AGE and their interaction with pathological hotspots of the disease. Furthermore, AGEP’s inhibition activity and inhibition in forming fructosamine have been attributed to biological interaction between pharmacologically active principles and functional groups (amino and carbonyl) of glucose molecules, which subsequently inhibits glycation reaction and the formation of AGEP. The protective effect of biologically active principles of AGE spares thiol and other functional groups of structural and functional proteins of cellular architecture provides AGE’s in-vitro anti-diabetic activity [40, 41]. Furthermore, there are a plethora of studies that have reported antioxidant activity of AGE to the presence of various antioxidants present in AGE; these findings are in agreement with GC-MS findings which report the presence of higher levels of diallyl disulfide, cis-2-ethyltetrahydro-3-methyl-thiophene, 2-methyl-5-(methylthio)-thiophene, 2-ethylthiacyclohexane, 4-ethylamine, 8-thiabicyclo [3.2.1] octane. Many studies have reported that these structural entities possess antioxidant activities [72–74].

In the current experimental design, we attempted to understand the molecular pathway involved in the pathogenesis of DM and the therapeutic mechanism of AGE in targeting potential hotspots involved in the progression of DM. DM was associated with a significant decline in intracellular Ca^2+^ levels through decreasing functionality of SERCA2, which reduces the secretory function of pancreatic β cells. Furthermore, some studies have postulated that chronic hyperglycemia stimulates pro-apoptotic pathways in pancreatic β-cells by activation of DR5/ caspase-8/3, which culminates in apoptosis of pancreatic β-cells, which further results in hyperglycemia-induced pro-apoptotic pathway and initiation of the vicious cycle of pancreatic β-cell degeneration and progression of DM. Our results indicate that AGE causes enhancement and preservation of pancreatic β-cell functioning and results in glucose homeostasis under basal and hyperglycemic conditions. These findings agree with earlier in-vitro studies that postulate that AGE causes improvement in β-cell functioning [75]. In line with these findings, our study indicates AGE supplementation has no significant effect on peripheral insulin resistance under normal conditions, and we could not observe any[76] considerable difference in insulin levels and body weight of laboratory animals in the AGE-DM and DM control group. Henceforth, these results concur with the findings of IPGTT and ITT, indicating that AGE exerts its therapeutic action of systemic glucose homeostasis by improving the functioning of pancreatic β cells.

Ca^2+^ homeostasis is tightly maintained by the endoplasmic reticulum (ER), and the release of insulin from pancreatic β-cells is interrelated with Ca^2+^ release from ER regulated by SERCA2. The cytosolic Ca^2+^ levels are pivotal for insulin secretion from β-cells as minimum threshold levels are required for exocytosis of insulin vesicles [75]. Our results indicate AGE causes the restoration of intracellular Ca^2+^ levels in pancreatic β cells by restoring the functioning of the SERCA2/Ca^2+^ pump, which subsequently results in the release of insulin from the pancreas. These findings are supported by [77], which reported that glucose metabolism inside β-cells results in the opening of voltage-gated Ca^2+^ channels in the plasma membrane and ER. An increase in cytosolic Ca^2+^ levels causes a further increase in intracellular Ca^2+^ levels by Ca^2+^ induced Ca^2+^ release from ER via SERCA2/Ca^2+^ pump, ultimately resulting in insulin release from pancreatic β-cells. It can also be postulated that AGE could interact with other biosynthetic pathways involved in the transcription/translation of insulin and exocytosis of insulin vesicles.

Furthermore, recent studies have found that persistent hyperglycemia causes ER stress, which deteriorates the quality of secretory proteins like pro-insulin, which further plays a role in the pathogenesis of DM. These cellular and subcellular changes cause misfolding of pro-insulin, which has been reported to cause activation of the pro-apoptotic pathway in a DR5-dependent manner, which results in the formation of caspase-8-activating complex and, ultimately, pancreatic β-cell death [78]. These findings are supported by postulates of [66], which demonstrated that DR5 causes activation of the “adaptor” protein Fas-associated death domain (FADD), which results in degeneration of ER and henceforth initiates cellular death [78].

SERCA2 activity has been studied in vertebrates and has been found to regulate endoplasmic reticulum (ER) Ca^2+^ levels. Any mutational change in structural characteristics of SERCA2 has been found to cause dysregulation in ER Ca^2+^ homeostasis, which subsequently causes improper functioning of various cellular subtypes [79]. Among the various mechanisms, SERCA2 has been postulated to play a pivotal role in the pathogenesis of DM by causing activation of pro-apoptotic pathways in pancreatic β-cells; SERCA inactivation causes accumulation of Ca^2+^ within ER which results in the activation of NADPH oxidase, which subsequently results in activation of NF-κB [80]. These mediators cause the activation of pro-inflammatory pathways and pro-apoptotic pathways/mediators, which results in cell proliferation, tissue fibrosis, and ECM deposition within pancreatic β-cells [81]. The elevated levels of these markers suggest DM is a low-inflammatory pathology, and the use of AGE’s anti-inflammatory and antioxidant activity holds a reliable, promising therapeutic approach in managing DM. TLR-4 is a macrophage pattern recognition receptor and is involved in the activation of NF-κB; recently, a vital link has been identified between the activation of TLR-4 and the pathogenesis of DM. Activation of TLR-4/NF-κβ causes activation of the pro-inflammatory pathway [82], and these findings are in concurrence with the results of the present study, where we observed substantial augmentation in TLR-4 and NF-κβ levels in diabetic animals.

Similarly, we observed AGE treatment in DM rats resulted in a significant decline in levels of TLR-4 and NF-κB, which indicates the pancreatic β-cell protective effect of AGE. The current study is a preliminary descriptive and correlational study; henceforth, further functional and mechanistic studies must be conducted to support our results. Furthermore, we propose in-depth studies on the therapeutic effects of AGE to understand its role and effective targeting of pathogenic hotspots.

## Conclusion

In summary, NF-kβ/ TLR-4 and SERCA/ Ca^2+^ pathways contribute to the pathophysiology of Type 2 Diabetes Mellitus (T2DM) by regulating β-cell functions and apoptosis. Our findings suggest that targeting these pathways could offer potential interventions for preventing and managing T2DM. AGE improves glucose homeostasis by activating pancreatic β-cell by restoring Ca^2+^ levels in pancreatic β-cells. AGE’s Selective activity was mediated through the NF-κB /TLR-4 pathway and SERCA/Ca^2+^ pathway. Likewise, the therapeutic benefits of AGE are mediated by the inhibition of pancreatic β-cell apoptosis, which subsequently causes an increase in serum levels of insulin, suggesting AGE as a viable alternative therapeutic approach for combating DM. The study implies that AGE may offer a natural and effective strategy for managing diabetes by targeting these pathways, potentially leading to improved glycaemic control and protection against diabetic complications. However, further research, including clinical trials, is warranted to validate these findings and determine the precise mechanisms underlying the observed effects of aged garlic extract in diabetes management.

## Data Availability

The data supporting this study's findings are available from the corresponding author upon reasonable request.
